# Characterization of a Field Spectroradiometer for Unattended Vegetation Monitoring. Key Sensor Models and Impacts on Reflectance

**DOI:** 10.3390/s150204154

**Published:** 2015-02-11

**Authors:** Javier Pacheco-Labrador, M. Pilar Martín

**Affiliations:** Environmental Remote Sensing and Spectrosocopy Laboratory (SpecLab), Instituto de Economía, Geografía y Demografía (IEGD), Consejo Superior de Investigaciones Científicas (CSIC), Albasanz 26-28, 28037 Madrid, Spain; E-Mail: mpilar.martin@cchs.csic.es

**Keywords:** spectroradiometer, automated system, characterization, sensor model, dark current, non-linearity, temperature dependence, spectral calibration, cosine directional response, hemispherical-conical reflectance factor

## Abstract

Field spectroradiometers integrated in automated systems at Eddy Covariance (EC) sites are a powerful tool for monitoring and upscaling vegetation physiology and carbon and water fluxes. However, exposure to varying environmental conditions can affect the functioning of these sensors, especially if these cannot be completely insulated and stabilized. This can cause inaccuracy in the spectral measurements and hinder the comparison between data acquired at different sites. This paper describes the characterization of key sensor models in a double beam spectroradiometer necessary to calculate the Hemispherical-Conical Reflectance Factor (HCRF). Dark current, temperature dependence, non-linearity, spectral calibration and cosine receptor directional responses are modeled in the laboratory as a function of temperature, instrument settings, radiation measured or illumination angle. These models are used to correct the spectral measurements acquired continuously by the same instrument integrated outdoors in an automated system (AMSPEC-MED). Results suggest that part of the instrumental issues cancel out mutually or can be controlled by the instrument configuration, so that changes induced in HCFR reached about 0.05 at maximum. However, these corrections are necessary to ensure the inter-comparison of data with other ground or remote sensors and to discriminate instrumentally induced changes in HCRF from those related with vegetation physiology and directional effects.

## 1. Introduction

Linking gas exchange measurements taken at single-point EC sites with spatial information provided by remote sensing is key to globally quantify and monitor the “breathing” of the planet [1]. However, the connection between these data sources is challenging due to the existence of spatial and temporal mismatches. Unattended ground-set optical sensors have the advantage of overcoming the temporal mismatch existing between the continuous micrometeorological measurements acquired by the EC systems and the periodic overpass of remote sensors. This way, information relative to the optical properties of vegetation can be directly related with the biospheric carbon and water fluxes, and used to upscale the flux information from site to local, regional and global scales [1,2]. Though the use of single or multi-spectral sensors at EC sites is more frequent due to their low cost and easy installation [3], hyperspectral sensors (spectroradiometers) are being gradually installed at these sites [2,3]. These sensors sample radiation in narrow and overlapping bands continuously arranged along the spectral domain, typically covering the visible and near infra-red (NIR) regions. On one hand, such detailed optical information can be related with the physiological and biochemical status of vegetation [4–11]; on the other it can be flexibly matched with the spectral bands of other remote sensors [12–14].

However, though information provided by spectroradiometers is rich and detailed, large uncertainties can affect the quantities of these spectral measurements [15,16]. This can be specially an issue in the case of unattended outdoor systems, which may face wide ranges of environmental conditions in terms of temperature, irradiance or illumination geometry, among others. These factors can produce changes in the radiometric quantities and in the computed reflectance factors which are not related with the optical properties of the target covers. Among the instrumental sources of uncertainty that can affect field spectroradiometers there are: the dark current, non-linearity, temperature dependence, spectral calibration or the directional response of the cosine receptors.

The dark current (DC) is a residual electrical current produced by a photosensitive device when this is not illuminated. It varies with the sensor's temperature (*T*) and the integration time (*t*_int_) [17]; which is the period while radiation is sampled in the sensor. The dark current is added to the photocurrent produced by the radiation, and its impact is larger the lower the photogenerated signal in the pixel.

The non-linearity (NL) is a variation in the proportionality between the radiance sampled and the output signal generated by the instrument. Non-linearity can be related with the gray level measured in each pixel; this makes less comparable the measurements taken under different radiation levels, measurements of targets of different bright, and for the different pixels of the array [18,19]. Moreover, non-linearity can be also related with the integration time when this is close to the sensor's readout time [20]. This artifact had been previously reported but not corrected in CCD cameras [20]; however a correction method valid for NMOS sensors was recently proposed by [21].

The energy bandgap of semiconductors, and therefore their photoresponse, is inversely related with their temperature. This phenomenon is known as temperature dependence (TD), and especially affects the near infra-red region in the case of silicon photodiodes [22,23]. Heat can also slightly modify the spectrometer dimensions and consequently the center and width of the spectral bands; the spectral range within each pixel is illuminated. The spectral calibration (SC), which is the function that relates the center of these bands with each pixel, can show thus a dependence on temperature. The accuracy and precision of SC is key when integrating information of different sensors or for fine resolution applications [24–26].

Finally, in the case of the sensors that sample hemispherical irradiance using cosine diffusers, the directional response of the cosine receptor (CR) can be also an issue. Ideally, the CR is the cosine of the illumination zenith angle (θ_s_); however, deviations from this behavior would introduce artifacts in the measurement of irradiance. The correction would require accounting for the fractions of diffuse and direct radiation in the environment [27].

Some of the abovementioned instrumental artifacts could be controlled, e.g., stabilizing the temperature of the instrument [9,11] but this might not always be possible. Also others are inherent to the instrument design so they cannot be prevented but should be characterized (e.g., non-linearity, or directional response of cosine receptors). Some of these artifacts have already been considered and corrected in automated systems in different ways [5,6,10,11,28], however to the best of our knowledge a full characterization accounting for all the factors identified in this work has not been previously reported.

The aim of this work is characterizing the instrumental responses of a field spectroradiometer integrated in an automated system currently installed in an EC site, to allow the correction of the Hemispherical-Conical Reflectance Factor [29]. This would help to improve the inter-comparison of data, the upscale of spectral information and the separation between observed changes in the optical properties of vegetation caused by instrumental factors from those directionally, phenologically, and physiologically induced.

## 2. Materials and Methods

### 2.1. Instrumentation

We describe the characterization of a commercial double beam field spectroradiometer (Unispec DC (SN 2038), PP Systems, Amesbury, MA, USA). This instrument allows calculating HCRF by simultaneously sampling upwelling (channel 2) and downwelling (channel 1) radiation. Channel 2 is a bare fiber optics (UNI685-6, PP Systems) whereas channel 1 is a fiber optics but with a cosine receptor (UNI686-6 + UNI435, PP Systems). Each channel is equipped with a Monolitical Miniature Spectrometer 1 (Carl Zeiss, Inc., Thornwood, NY, USA), composed by a fixed grating and a silicon diode array S8381-256Q NIR-enhanced sensor (Hamamatsu Photonics K.K., Tokyo, Japan). The Unispec DC operates in the Visible-NIR (300–1100 nm) with a radiometric resolution of 16 bits, a nominal bin size of 3.3 nm and <10 nm of spectral resolution (Full Width at Half Maximum, FWHM). The instrument does not have shutters to automatically record dark current but provides temperature readings through a temperature sensor inside the spectroradiometer which can be used to model DC [28].

This spectroradiometer has been installed in the field as the core instrument of an AMSPEC system [28,30], in the Majadas del Tiétar FLUXNET site (www.fluxnet.ornl.gov), Cáceres, Spain (denominated AMSPEC-MED system). These systems can continuously sample canopy spectra at different viewing and illumination angles in order to characterize the canopy BRDF and estimate light use efficiency (LUE) and other biophysical variables [4,7,12]. The AMPSPEC-MED system is powered by solar panels. Power constrains do not allow stabilizing the instrument temperature as in other unattended systems [9,11], thus fans are used instead when air temperature goes over 30 °C.

In the EC site, diffuse-to-global radiation ratios (*DGr*) are continuously measured and integrated every ten minutes by a SPN1 Sunshine Pyranometer (Delta T Devices, Cambridge, UK). This instrument samples global and diffuse irradiance between 400 nm and 2800 nm. As in our study spectral *DGr* is needed to correct CR, this has been modeled using an ASD Fieldspec 3 spectroradiometer (Analytical Spectral Devices Inc., Boulder, CO, USA), with a spectral range (350 nm to 2500 nm) close to the pyranometers's one, and a calibrated Spectralon^®^ panel (Labsphere Inc., North Sutton, NH, USA).

Prior to field deployment, the Unispec DC was characterized in the Environmental Remote Sensing and Spectroscopy Laboratory (SpecLab-CSIC, Spain). As in the AMSPEC-MED, the Unispec DC User Interface Computer was bypassed and the instrument was controlled through a RS-232 connection using a fit-PC2i computer (CompuLab, Yokneam, Israel); and controlled using a Matlab routine [12,28]. For the characterization in the laboratory, an ASD RTS-3ZC integrating sphere (Analytical Spectral Devices Inc.) was used as homogenous light source. The sphere's inner surface coating is highly reflective (>95% Zenith Polytetrafluoroethylene (PTFE), Sphereoptics Hoffman LLC, Contoocook, NH, USA); and it is illuminated by a 10 W quartz-tungsten-halogen bulb powered by a stabilized source. A collimated beam is sent through one of the ports of the sphere and reflected by a 99% Zenith PTFE in front of it. Radiation is scattered in all directions and measured through a second open port, normal to the collimated beam, where both Unispec DC fiber optics are aimed.

A mercury-argon calibration source (Ocean Optics, Dunedin, FL, USA) and a 250 W quartz-tungsten-halogen bulb irradiance source (OSRAM GmbH, Munich, Germany) have been used for spectral calibration and the characterization of the cosine receptor directional response respectively. The spectroradiometer temperature was regulated using a drying oven Raypa DOD-90 (R. Espinar, Terrasa, Spain) and a fridge CTP 31213 (Lieberh, Ochsenhausen, Germany).

### 2.2. Experimental Setup

This section describes the experiments and measurements carried out in order to characterize the Unispec DC spectroradiometer responses to under different environmental conditions and instrument settings.

#### 2.2.1. Dark Current

DC was characterized as a function of the integration time and the sensor's temperature. The optical fibers connected to the instrument were covered to block the entrance of light. Like in the other experiments described in this work, where the instrument temperature was modified, the spectroradiometer was first cooled down in the fridge. Then the experiment started and measurements were done while it was warmed up in the oven. Once it reached a maximum temperature, the instrument was cooled down at environmental conditions, while a second set of measurements was taken. In the cold down experiments the instrument never reached temperatures as low as those used at the beginning of the warm up since cooling was not forced. This way, two different models were adjusted both for the warm-up and the cool-down processes. DC measurements started when sensor temperature was 9.5 °C, when it reached 45.4 °C the instrument was cooled down to 24.2 °C. Meanwhile measurements were continuously taken, randomly varying the *t*_int_ between 4 ms and 1000 ms (Table 1). Similarly as the instrument operates in the field, the integration time was first set and then the number of scans averaged was selected so that the full measurement took a time equal or shorter than 2 s. This configuration was also applied to the others experiments.

#### 2.2.2. Non-Linearity

For the NL characterization, the spectroradiometer was warmed up at environmental temperature until the sensor's *T* was stable (notice that the range presented in Table 1 is due to random noise of the *T* sensor). Optical fibers for channels 1 and 2 were aimed into the integrating sphere port. Ten measurements of the radiance source were taken at 40 different and randomly selected integration times, ranging between 4 ms and 741 ms (Table 1).

#### 2.2.3. Temperature Dependence

The temperature dependence of the Unispec-DC was characterized by collecting measurements while the instrument was warmed up and cooled down. In this experiment, *T* started at 13.9 °C and was increased up to 46.1 °C; then the instrument was cooled at environment temperature up to 25.6 °C. In this case, the optical fibers were also aimed into an open port of the integrating sphere. Measurements were continuously acquired at four different integration times (Table 1); every time that a new integration time was set five spectra were taken.

#### 2.2.4. Spectral Calibration

The spectral calibration experiment was repeated at different temperatures in order to assess any significant influence of *T*. In this case the instrument was warmed up from 15.6 °C to 48.3 °C and then cooled to 18.3 °C. We alternately plugged the optical fibers of channels 1 and 2 into the Hg-Ar source and took ten measurements using always the same integration time.

#### 2.2.5. Cosine Receptor Directional Response

The cosine receptor directional response was characterized by rotating the Unispec DC's cosine receptor in front of a fixed light source between 0° and 90°, at 10° steps. The experiment was done twice, rotating the cosine head 90° over its central axis in order to acquire measurements at different zenith angles in two perpendicular planes of the cosine head. The experiment was carried out in a dark room in order to minimize diffuse radiation. In each position of the cosine receptor, five measurements of the global radiation were acquired first, then the cosine diffuser was shaded using a small opaque plate, and five measurements of the diffuse radiation were recorded. During the experiment the sensor temperature was stable, ranging randomly between 26.4 °C and 29.3 °C.

#### 2.2.6. Diffuse-to-Global Radiation Ratio

Spectral *DGr* is needed for applying the CR correction. However, quite typically diffuse-to-global radiation ratio is only provided by broadband meteorological sensors in the field (*DGr*_broadband_). In this study we use an ASD Fieldspec 3 spectroradiometer to model spectral DGr and *DGr*_broadband_ by acquiring irradiance measurement under different sky conditions between 350 and 2500 nm. Modeled ratios are later used to predict the spectral *DGr* in the Unispec DC from the broadband measurements of the SPN1 sensor in the field. Global irradiance was measured using a calibrated Spectralon^®^ panel, whereas diffuse radiation was measured shading the same panel with an opaque plate alternately [31]. High zenith angles were avoided to minimize the effects of panel anisotropy [31]. Since diffuse and global measurements were not simultaneous, global irradiance was linearly interpolated to the timestamps of the diffuse measurements. Then *DGr* was calculated by dividing the diffuse irradiance by the interpolated global irradiance.

### 2.3. Sensor Models

This section describes the models adjusted to the experimental data that describe the responses of the sensor to radiation as a function of the different variables modified during the experiments.

#### 2.3.1. Dark Current

The dark current can be characterized as a variable proportional to the integration time and quadratically dependent of the temperature [17]. In addition, during the experiment we found a negative trend of the measured dark signal (*N*_dark_) with the temperature at low integration times. This suggested that the recorded spectra could be actually composed by electrons thermally generated in the photodiode (*N*_0_) plus an electronic bias (*N*_bias_) inversely proportional to temperature [21,32,33]. Consequently we characterized *N*_dark_ as the addition of both signals, as described in Equation (1). Coefficients *a* and *b* in Equation (1) were fitted per each pixel (*i*) of each Unispec-DC channel by using ordinary least squares regression. Hysteresis [17] was accounted for by fitting one model for the warm-up and another for the cool-down processes separately:

(1) $$N_{\text{dark},i}=N_{\text{bias},i}(T)+N_{0,i}(t_{\text{int}},T)=(b_{0,i}+b_{1,i}T)+t_{\text{int}}\cdot (a_{0,i}+a_{1,i}T+a_{2,i}T^{2})$$

For every spectrum *N*_bias_ was first removed so that the measured signal (*N*_meas_) later processed is the addition of the signals produced by the photocurrent (*N*_phot_) and *N*_0_.

#### 2.3.2. Non-Linearity

In field spectroradiometers non-linearity is usually characterized as a function of the gray level measured [34]. However, a second source of non-linearity has been found in this instrument and has been characterized using a new methodology; a complete description can be found in [21]. This method characterizes the responses of the instrument to both sources of non-linearity simultaneously from the measurements of a single experiment. The second NL is related with the integration time, and is described as a leakage of electrons from the pixel to the output line during the readout phase [20]. This is represented by the function ℜ_IT_ (Equation (2)), where linearity is proportional to the total amount of electrons leaked during the readout phase in each pixel (*B_i_*) divided by the integration time set. In [21] was also shown that *B_i_* increases with the radiance in the pixel until a maximum level. Since this instrument has not a radiance calibration, *B_i_* is defined as a function of a variable named “instrumental radiance” (Equation (3)), which is proportional to radiance after gray level-related non-linearity has been corrected using ℜ_GL_. This variable, L*_measGLcor,*i*_, is calculated dividing *N*_meas_ by ℜ_GL_ and *t_int_*:

(2) $${ℜ}_{\text{IT},i}=1+\frac{B_{i}({L^{*}}_{\text{measGLcor},i})}{t_{\text{int}}}$$

(3) $$B_{i}=C-\frac{D}{{L^{*}}_{\text{measGLcor},i}}\cdot \log\left(1+\frac{{L^{*}}_{\text{measGLcor},i}}{D}\right)$$

#### 2.3.3. Temperature Dependence

Temperature dependence was characterized normalizing the sensor responses by the sensor responses measured at a given temperature. To avoid the influence of other variables, DC and NL corrections were first applied to *N*_meas_ resulting *N*_phot_. These corrections are later described in Section 2.4. TD was then calculated as the ratio between *N*_phot_ measured at different *T* and the *N*_phot_ linearly interpolated to a reference *T* arbitrarily selected (30 °C) as in Equation (4). In order to minimize the impact of noise in the pixels where signal-to-noise ratio was low, TD was smoothed with a robust local regression using weighted linear least squares (RLOWESS) method [35]. A 5th degree polynomial was fit for each pixel of each sensor relating TD and *T*:

(4) $$TS_{i}=\frac{N_{\text{phot},i}}{N_{\text{phot},i_{30{}^{\circ}C}}}$$

#### 2.3.4. Spectral Calibration

Experimental data were first corrected using the sensor models previously described and the method described in the Section 2.4. Then spectral calibration measurements were used to fit a second degree polynomial that assigns wavelength units to the pixels of each sensor. This polynomial included also a temperature factor to account for the temperature related spectral shifts. In the spectra recorded several emission lines whose wavelength is known were selected. The center of the emission lines was calculated as the mean of a normal distribution fit on each selected emission line of the spectra; however, if the coefficient of determination was lower than 0.9, these emission lines were discarded. Eventually, only the emission lines that remained were used to adjust the model.

#### 2.3.5. Cosine Receptor Directional Response

Prior to any other calculation, measurements were corrected as described in Section 2.4 using the models previously adjusted. CR was characterized using exclusively the direct radiation measured during the experiment. Therefore, residual diffuse radiation was subtracted from global radiation to characterize the cosine response using only direct radiation. CR was characterized as the ratio between the direct radiation measured at each angle normalized by the direct radiation at nadir. As defined in Equation (5), a correction factor β*_i_*(θ_s_) was calculated as the difference between the cosine of the illumination angle and CR [27]. In each pixel, a 7th degree polynomial was fit to model the correction factor β*_i_* as a function of θ_s_:

(5) $$\beta_{i}(\theta_{s})=\cos(\theta_{s})-\overset{N_{\text{phot}\;\text{direct},i}(\theta_{s})}{\;}/\underset{N_{\text{phot}\;\text{direct},i}(0)}{\;}$$

#### 2.3.6. Diffuse-to-Global Radiation Ratio

The spectral *DGr* in each band of the channel 1 of the Unispec DC was modeled as a function of the broadband *DGr* measured in an EC tower by a single-band SPN1 Sunshine Pyranometer. For that, the *DGr*_broadband_ was simulated from the global and diffuse irradiances measured with the ASD Fieldspec 3 integrating the spectral irradiance between 400 and 2500 nm weighted by the nominal spectral response of the SPN1 sensor [36]. The 283 *DGr* spectra generated were resampled to the spectral bands previously estimated for the channel 1 of the Unispec DC using the spectral convolution method [37] and the nominal spectral resolution of the instrument, 10 nm. The Unispec DC itself was not used since the *DGr* measurements acquired with the cosine receptor would have been affected by the directional response of the diffuser, for whose correction the *DGr* is needed. Then the simulated spectral *DGr* of each pixel of the Unispec DC (*DGr_i_*) was characterized as a linear function of the simulated *DGr*_broadband_ (Equation (6)):

(6) $$DGr_{i}=a_{0,i}+a_{1,i}\cdot DGr_{\text{broadband}}$$

### 2.4. HCRF Correction

**Scheme 1.**
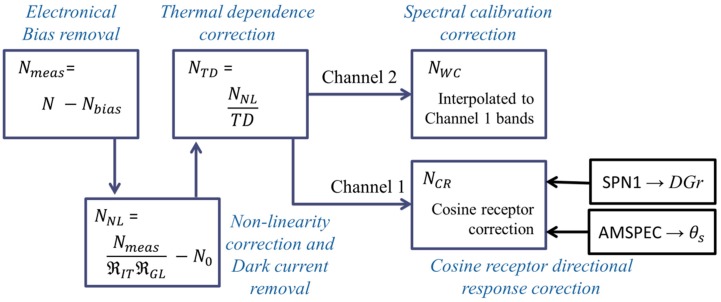
Summary of the corrections performed to the spectral data acquired by the Unispec DC spectroradiometer.

We used the described sensor models adjusted in the laboratory in order to correct spectral measurements provided by the Unispec DC integrated in an outdoors automated system. A summary of the corrections applied is shown in the Scheme 1; any spectra quantified in digital numbers units, at different stages of the correction, is represented in a general way by the variable *N* and a subscript related with the correction level.

In this paper, we present the corrections applied to the spectra acquired by the AMSPEC-MED system in a single viewing position between the 1 August 2013 and the 15 June 2014. Spectra were taken with a viewing azimuth and zenith of 190° and 40° respectively. Saturated and corrupted spectra were removed and eventually 3730 measurements were selected. For this dataset, sensor's temperature ranged between 1.2 °C and 44.4 °C, integration time was set between 8 and 4000 ms, θ_s_ ranged between 16.8° and 77.8°, and *DGr*_broadband_ integrated every ten minutes by the SPN1 sensor ranged between 0.063 and 0.986. All the spectra were originally acquired in raw digital numbers (DN) for each channel.

The correction began estimating *N*_bias_ and *N*_0_ from Equation (1) so that *N*_bias_ was subtracted from the original digital numbers to obtain *N*_meas_. Then NL correction was applied to those pixels where the signal was larger than *N*_0_, which was removed afterwards (Equation (7)); this way, *N*_NL_ was calculated. After that, we estimated TD for each pixel as a function of *T*, and normalized *N*_NL_ to the sensor's response at 30 °C (Equation (8)), resulting *N*_TD_. Next, since the spectral calibration of each channel is different, we resampled *N*_TD_ spectra of channel 2 to the center bands of channel 1 using linear interpolation (*N*_SC_). For those corrections where two models (warm-up and cool-down) had been calibrated, the daily trend of temperature was used to decide which model would be used:

(7) $$N_{\text{NL},i}=\frac{N_{\text{meas},i}}{{ℜ}_{GL}\left(N_{\text{meas},i}\right){ℜ}_{\text{IT}}\left(t_{\text{int}},{L^{*}}_{\text{measGLcor},i}\right)}-N_{0,i}$$

(8) $$N_{TD,i}=\overset{N_{NL,i}}{\;}/\underset{TD_{i}(T)}{\;}$$

Finally, we applied the CR correction to *N*_TD_ spectra of channel 1 following the methodology described in [27]. First, we linearly interpolated the *DGr*_broadband_ (integrated every 10 min by the SPN1) to the timestamp of each spectrum. Then we used the interpolated values to estimate the *DGr_i_* in each spectral band using the previously adjusted model (Equation (6)). The sun zenith angle is calculated by the AMSPEC routine [38] and provided with the spectra metadata [4]. The correction factor β*_i_* and the *DGr_i_* are used to correct the instrumental irradiance as defined in Equation (9):

(9) $$N_{CR,i_{Ch\;1}}=N_{TD,i_{Ch\;1}}\cdot \left[1-DGr_{i}\cdot \int_{1}^{0}\beta_{i}(\theta_{s})\theta_{s}d\theta_{s}-(1-DGr_{i})\cdot \beta_{i}(\theta_{s})\right]$$

Finally, reflectance is calculated using the cross-calibration method [6], where the channels' ratio is corrected using the measurement of a calibrated white reference panel (while channel 1 measures downwelling irradiance) as in Equation (10), where *ρ*_Reference_ is the absolute reflectance correction factor of the reference panel:

(10) $$\rho_{\text{DBM},i}=\frac{{\left(N_{WC,i_{Ch\;2}}\right)}_{\text{Target}}}{{\left(N_{CR,i_{Ch\;1}}\right)}_{\text{Target}}}\cdot \frac{{\left(N_{CR,i_{Ch\;1}}\right)}_{\text{Reference}}}{{\left(N_{WC,i_{Ch\;2}}\right)}_{\text{Reference}}}\cdot \rho_{\text{Reference},i}$$

## 3. Results and Discussion

### 3.1. Dark Current

Measured dark signal ranged between 81 and 829 and between 99 and 797 DN in channels 1 and 2 respectively. At low *t*_int_ we observed a negative trend of the dark signal with *T*, which led us to model *N*_dark_ as defined in Equation (1). Errors in the fitting were low, Relative Root Mean Squared Errors (RRMSE) in the warm-up and cool-down models were 2.83% and 3.45% in channel 1 and 2.53% and 4.46% in channel 2 respectively.

**Figure 1.**
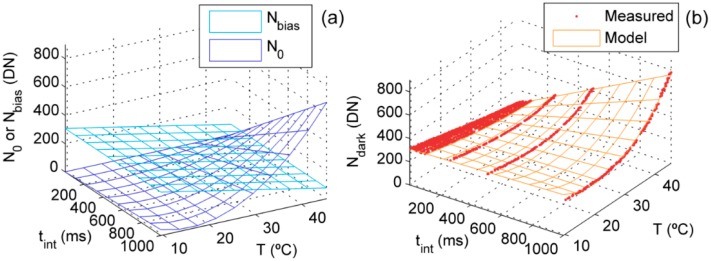
Channel 1's dark signal models in pixel 170 of sensor while warming up: (**a**) Modelled dark current (*N*_0_) and electronic bias (*N*_bias_); (**b**) Modelled and measured dark signal (*N*_dark_).

Figure 1a separately depicts the modeled *N*_0_ and *N*_bias_ in a pixel of channel 1 predicted by the warm-up model. As shown, *N*_bias_ linearly decreases with *T* and *N*_0_ is weaker than *N*_bias_ at low temperatures. Figure 1b shows the predicted and measured *N*_dark_ for the same pixel; as can be seen, the dark signal increases with the temperature at large integration times, and decreases at low integration times.

### 3.2. Non-Linearity

**Figure 2.**
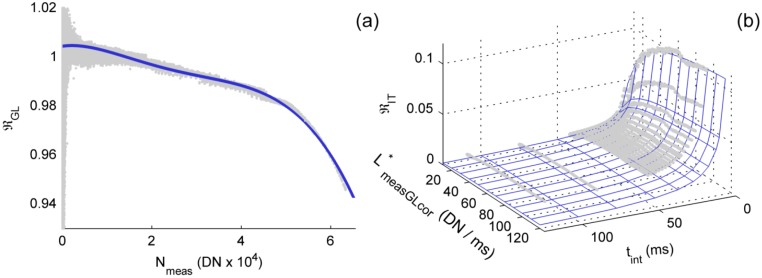
Channel 1's non-linearity models: (**a**) NL model related with the gray level (ℜ_GL_); (**b**) NL model related with the integration time (ℜ_IT_).

NL measurements covered the full sensor's radiometric range and also used very low *t*_int_; this allowed adjusting the models ℜ_GL_ and ℜ_IT_ [21]. Figure 2 shows the predicted and measured values for each one of the corrections functions corresponding to channel 1.

Figure 2a depicts ℜ_GL_, which slightly decreases with the gray level measured up to dropping above 50.000 DN. RRMSEs of the fit were 0.30% and 0.40% in channels 1 and 2 respectively. Figure 2b shows the predicted and measured values of ℜ_IT_, in this case RRMSEs were 0.22% in channel 1 and 0.24% in channel 2. As can be seen, ℜ_IT_ asymptotically increases with L*_measGLcor_ up to a maximum value at low *t*_int_, and drops quickly as the *t*_int_ increases.

### 3.3. Temperature Dependence

**Figure 3.**
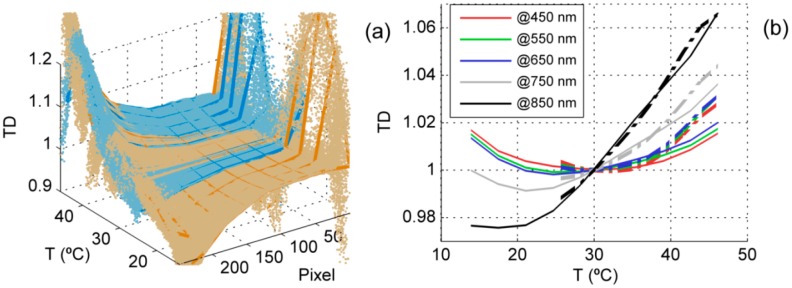
Temperature dependence models in channel 1: (**a**) Warm-up model and data (in orange)cool-down model and data (in blue). (**b**) Warm-up (thin solid lines) and cool-down models (thick dashed lines) for pixels close to different wavelengths.

Measured TD ranged between 0.90–1.21 in channel 1 and 0.86–1.19 in channel 2 (95% confidence). Pixels in the extremes of the sensors, especially in the ultraviolet region, were very noisy due to the low signal. Figure 3a shows the adjusted models in channel 1. As can be seen, the sensitivity of the sensors varied with the temperature, especially in those pixels corresponding to the largest wavelengths, above pixel 120 (∼700 nm), where the sensitivity increased with *T*. Predictive models were precisely fit, though noise was large in the extremes of the sensor array. Between 400 nm and 1000 nm RRMSEs for the warm up and the cool down models were 0.155% and 0.094% in channel 1 and 0.160% and 0.087% in channel 2 respectively. Figure 3b shows the hysteresis of temperature dependence for different pixels.

### 3.4. Spectral Calibration

**Figure 4.**
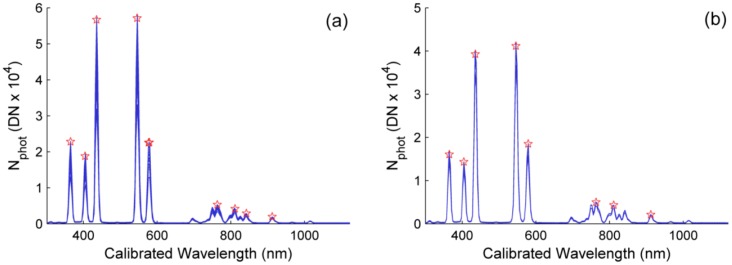
Hg-Ar lamp emission lines spectra. The bands selected for the spectral calibration of each channel are marked with a star. (**a**) Channel 1; (**b**) Channel 2.

In order to locate the center of the emission peaks in the sensor array, a normal distribution function was fit to the emission lines of the Hg-Ar lamp. However, for the spectral calibration, only those emission lines where correlation coefficient of the fit was high were used. This way, nine and eight lines were selected for channel 1 and channel 2 respectively (Figure 4). Center band position showed a small decreasing trend with *T*, with slopes ranging between −0.0048 and −0.0006 nm/°C. For each sensor a second order polynomial was fit relating a center wavelength to each pixel of the array. The effect of temperature was tested including this variable in the models. Differences found between the wavelengths predicted by each model ranged between −0.081 nm and 0.075 nm in channel 1 and −0.098 nm and 0.094 nm in channel 2. Therefore, considering the spectral features of the sensors and the noise of the temperature readings, *T* was not included in the spectral calibration models. A second degree polynomial was fit relating the pixel position and the corresponding spectral band; Root Mean Squared Errors (RMSE) of the models were 0.920 nm and 0.714 nm in channel 1 and 2 respectively. RMSEs were larger in the NIR, where emission lines were wider and noisier than in the visible. Spectral ranges estimated for channels 1 and 2 respectively were 301.13–1122.98 nm and 300.10–1122.16 nm.

### 3.5. Cosine Receptor Directional Response

**Figure 5.**
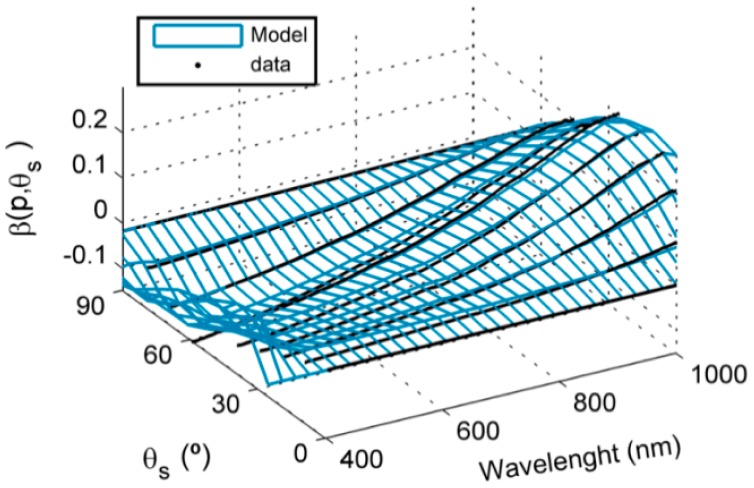
Cosine receptor directional response correction factor β(θ_s_). Fit model and measured data.

Figure 5 shows the correction factor β*_i_*(θ_s_) calculated as the difference between the ideal and the measured cosine response.

The cosine receptor overestimated irradiance at wavelengths lower than 700 nm. This threshold shifted to above 850 nm as the illumination angle increased. Maximum differences from an ideal cosine response were between −0.156 and 0.169 in the range 400–1100 nm, and were largest at the middle angles, around 60°. A polynomial model was fit for each pixel with an overall RRMSE of 1.03%. Diffuse radiation fraction was lower than 2.14% in the range 400–1000 nm with the illumination at nadir.

### 3.6. Diffuse-to-Global Radiation Ratio

**Figure 6.**
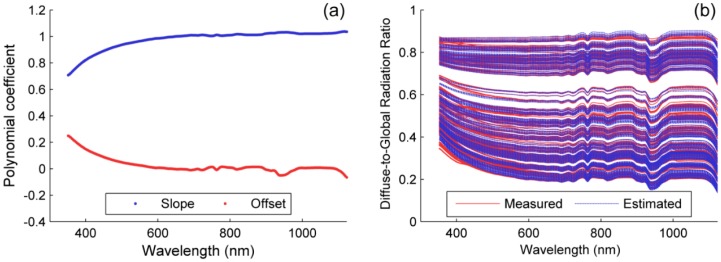
(**a**) *DGr* linear model coefficients. (**b**) Measured and estimated spectral *DGr*.

For each band of the channel 1, a linear model was fit to predict the spectral *DGr* from the *DGr*_broadband_. Figure 6a shows the slope and the offset of each model and Figure 6b depicts the measured and the modeled *DGr*. As can be seen, spectral *DGr* is lower in the atmospheric absorption bands; these features are noticeable in the offset of the models, which decrease from the Visible to the NIR. On the contrary, the slope of the models increases towards the NIR. Mean RRMSE in the prediction of spectral *DGr_i_* was 1.21%.

### 3.7. HCRF Correction

**Figure 7.**
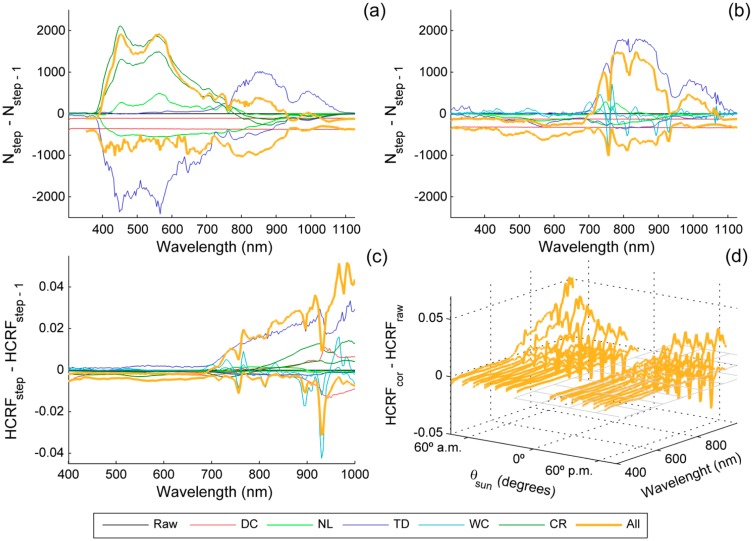
HCRF correction performed on 3730 spectra taken by the AMSPEC-Med system between the 1 August 2013 and 15 June 2014 in a single viewing position. Percentiles 99% of the changes introduced by each step of the correction respect to the previous stage are shown: (**a**) DN spectra in channel 1; (**b**) DN spectra in channel 2; (**c**) HCRF spectra; (**d**) HCRF spectra grouped in 10 degrees wide ranges of θ_s_.

In order to assess the influence of each correction both on the digital numbers and the reflectance factor spectra, we corrected a dataset of measurements acquired by the AMSPEC-MED system for almost ten months, under very different environmental conditions. The changes introduced by each step of the correction (Scheme 1) respect to the previous step were analyzed; and also the differences between the raw and the completely corrected values are also calculated. Results of this analysis are summarized in Figure 7, where the 99% confidence intervals of the changes introduced in this dataset are shown. Figure 7a,b show the differences observed in the DN spectra (N) of channels 1 and 2 respectively. In this figure we have merged the removal of the dark current and the electronic bias in order to assess independently the impact of the dark signal. However, it must be noticed that *N*_bias_ is removed in the first step of the correction whereas *N*_0_ is removed after the non-linearity correction (Scheme 1). As can be observed, in channel 1, the largest changes in *N* were produced by the temperature dependency correction. These were mainly negative in the Visible region, and became more clearly positive in the NIR. CR corrections also introduced large variations, with positive differences below 780 nm, and negative above 800 nm. Non-linearity correction produced changes in *N* of lower magnitude than TD and CR, which where related with the measured DN value when positive. All the corrections applied together led to increases and decreases of *N*. The increases were larger than the decreases in the visible region and decreases were larger in the NIR. In channel 2 the temperature dependence correction also produced the largest variations; these were maxima above 700 nm with a positive effect. NL correction mainly produced decreases of DN, though increases were registered between 710 and 860 nm, where the signal was also maximum. The spectral calibration correction led to irregular differences that peaked around the atmospheric absorption features. These were maximum around 756 nm, close to the atmospheric O_2_-A absorption band. In the overall, corrections in channel 2 produced a decrease of *N* in the Visible region and increases and decreases in the NIR, where the first were of larger magnitude. In both channels, DC correction produced a moderated decrease in *N*.

Figure 7c similarly shows the changes introduced in HCRF by each correction. HCRF calculation is limited to the spectral region between 400 to 1000 nm due to the noise found in the models out of this range. DC correction slightly modified the HCFR, producing small decreases below 735 nm and larger differences, both positives and negatives, above this wavelength. NL produced small decreases of HCRF in all the spectral range, but in the range 720 nm to 900 nm, where some increases were also registered. TD correction produced small changes below 700 nm in HCRF, but above this point, large increases were registered. SC correction produced peaky changes around the atmospheric absorption bands, mainly located in the NIR such as 760 nm (O_2_-A) and 820 nm, 930 nm and 970 nm (H_2_O). Variations introduced by the CR corrections were small and negative in the Visible; increases became larger and positive above 700 nm and were always positive above 800 nm. All the corrections together led to small decreases in HCRF between 400 nm and 700 nm, but these became larger and also positives in the NIR region. Figure 7d depicts the percentile 99% of changes introduced by all the corrections in HCRF grouped in different moments of the day. The dataset has been classified using ranges of θ_s_ with a width of 10 degrees and the time of the day (a.m. or p.m.). As can be seen, the effects of the corrections are larger the larger is θ_s_. The differences between the corrected and non-corrected HCRF were minimum at noon and larger in the morning than in the afternoon for the same elevation angles.

## 4. Discussion

Different instrumental sources of error in the computation of HCRF have been characterized in laboratory under different temperatures and configuration settings. The Unispec DC spectroradiometer is not provided with a shutter that allows measuring dark current, however, it can be retrieved as a function of *t*_int_ and *T*. Hysteresis can be accounted for by using the *T* variations along the day to establish when the instrument is warming up or cooling down. Temperature ranges used were shorter for cooling experiment than for the warming up experiments, since cooling could not be controlled. However, models adjusted are still suitable to correct field data, since this is similar to what actually occurs outdoors; temperatures in the morning are lower than temperatures at the end of the afternoon. A similar characterization performed on a MMS 1 spectroradiometer also found quadratic and linear relationships between dark current and *T* and *t*_int_ respectively [17]. In that case, minimum *t*_int_ were larger than the ones used here, and the Front End Electronics that controlled the spectrometer was different; thus the presence of a bias or a negative trend like the one we found could not be compared. The authors have not a clear explanation for the reported bias inversely related with the temperature, but it might be explained by a change in the capacitance of the condensers related with the temperature. Though both *N*_bias_ and *N*_0_ are eventually subtracted from the spectra, the separation is necessary for the correction of NL.

The non-linearity correction method we used proved having a better performance during independent validations [21], and would provide corrections more reliable than other methods under AMSPEC-MED operating conditions. This method corrects non-linearity using the functions ℜ_GL_ and ℜ_IT_; the first of them is related with the electronics that process the analogical signal read from the sensor, and the second with a leakage of photocurrent generated during the readout. The AMSPEC-MED system automatically sets the *t*_int_ of each spectrum so that the signal in channel 1 reaches about 40,000 DN, this prevents reaching very high values, where the influence of ℜ_GL_ is large. However, *t*_int_ set under sunny conditions are low and measurements could be potentially affected by photocurrent leakage. The effects of non-linearity in HCRF are, however, lower than those produced by other corrections. This can be explained on one hand because the *t*_int_ auto-adjustment is designed to keep measurements within a range of DN values where of ℜ_GL_ is low. On the other hand, the *t*_int_ is the same for both channels and both also reach high “instrumental radiances” (in DN/ms) [21]. Since the electron leakage (*B_i_*) rapidly increases at low radiance levels, approaching asymptotically a maximum value (Figure 2b), ℜ_IT_ would be similar in channels 1 and 2 too, and would cancel mutually when DN spectra from these channels are divided to calculate HCRF (Equation (10)). However this cancellation might not occur in all the cases; for example, when measuring shaded targets the upwelling radiation channel might register low instrumental radiances, or in the case of sensors with a high spectral resolution, within atmospheric absorption lines. In that case, ℜ_IT_ could be different in each channel, leading to artifacts in HCRF, and also in LUE estimators as those derived from spectral indices such as the Photochemical Reflectance Index (PRI) [39] or Sun Induced Fluorescence [40].

Within the range of temperatures registered during its characterization, the temperature dependency normalized at 30 °C varied less than 0.032 between 400 and the 700 nm, but above this region, variation exponentially increased up to 0.23 at 1000 nm. This might be explained due to proximity to the band edge of the silicon, which is sensible to *T* [19]. Compared with the other corrections the temperature dependence produced large changes in HCRF, especially in the NIR region. Unlike in the case of non-linearity, differences between both channels did not seem to cancel out during the calculation of the reflectance factor. Changes introduced in the raw DN spectra were also large, and might be significant in the quantification of the measured radiation.

The spectral calibration showed a dependency on temperature; however, the magnitude of these observed drifts compared with the spectral characteristics of the sensor and the model errors suggested that it could be overlooked. Actually, the inclusion of *T* on the calibration models barely produced any difference in the calibrated wavelengths and was eventually removed. Spectral resolution and sampling interval of the Unispec DC are suitable for the characterization of vegetation reflectance and computation of different vegetation indices [6,41,42]; however, it cannot be used for other applications which require very high spectral resolution, such as sun induced fluorescence retrieval [40,43]. Though we discarded the influence of *T* on the spectral calibration, this might be still considered for instruments whose applications require very high spectral accuracy. Though we were able to characterize the spectral shift, we could not measure the changes in the FWHM. However, considering the small shifts in the center wavebands in the case of this instrument and its application, we assumed that these should be also negligible.

The characterization of the cosine receptor's directional response allows correcting the downwelling radiation spectra taking into account the direct and diffuse fractions. [44] empirically inferred spectral irradiance from a reduced number of spectral bands; though estimations would not be reliable under passing clouds. A simple approach has been used here, since *DGr* is measured with a single broadband pyranometer in the EC site, this single value is used to infer the spectral *DGr*. The modelling was done using an ASD spectroradiometer with a spectral range slightly narrower than the one of the SPN1 sensor; however, irradiance in the spectral range not measured by the ASD is low, and should have little effect on the model. CR corrections rely also on the directional responses of the Spectralon^®^ panel used for the modelling and the directional response of the SPN1 sensors. For this reason, large θ_s_ were avoided during the modelling of the *DGr* to minimize directional dependencies on the panel. Moreover, the directional response of the cosine receptor used in the AMSPEC system is known to be further from the ideal response when compared with the responses of other cosine heads [45]. For these reasons, we expect that this correction is able to improve the quality and inter-comparability of data. The CR correction applied relies on the assumption that the diffuse skylight is isotropic, and the *DGr*_broadband_ provided is the average of ten minutes period; this can lead to uncertainties under heterotrophic and unstable sky conditions. However, data used to separate physiological from directional changes in PRI [4] must be acquired under similar illumination conditions. Thus, unstable conditions would force to reject these data even if *DGr* measurements were available all the time. Nonetheless, this correction introduced large changes both in the *N* and HCRF spectra; thus, CR correction should be regularly included and further research should be done to achieve reliable estimates of *DGr_i_*. CR correction produced the second largest changes in the *N* spectra of channel 1, and also in the HCRF in the NIR.

The analysis of the effects of all the corrections across the day (Figure 7d) can be related mainly with two corrections. On one hand, the CR correction produces larger differences in the NIR region the larger is θ_s_, since in this region the directional response is furthest from the cosine response than in the Visible. Moreover, the largest changes occur during the morning, this can be related to the temperature dependence correction. Due to power constrains, the instrument is not thermally stabilized, and starts operating as soon as it switches on in the morning. The largest changes of temperature are thus experimented in the first hours of the day, during which the instrument is warmed up by the circulating power and by the increasing environmental temperature. *T* varies less during the afternoon, when is stable and slightly decreases at the end. Thus, the temperature dependence correction, which is based on a reference of 30 °C, produces large changes in the NIR region during the first hours of operation.

The effects observed are limited to the spectroradiometer used for this work. Nonetheless, the instrument has been tested under a wide range of environmental conditions, showing how instrumental issues can operate and modify the measurements. Results suggest that similar characterizations should be applied to spectroradiometers integrated in outdoors unattended systems. Since characteristics of each instrument would be unique and the requirements of each application also different, the selection of which sensor models should be used and how should be experimentally adjusted could vary. However, some of the methods proposed in this work could be either directly used or adapted to characterize other instruments. Additionally the reported impacts of each model on the spectral variables considered can help other users to take decisions about systems development, instrument configuration and data analysis. Corrections applied generated differences in HCRF close to 0.05 at maximum, partially due to cancelling effects and partially due to the configuration of the system. The largest changes occurred in the NIR bands, which are of relevance in the study of vegetation vigor, structure and sun induced fluorescence. Effects in the Visible bands were much lower, however, this not might happen all the times, depending on the instrument characteristics, configuration and targets measured. Effects on the quantification of radiance or irradiance would be related with the changes introduced in the digital numbers by the different corrections. However, the instrument lacks of radiometric calibration, and these could not be assessed. Moreover, maintenance of updated sensor models shall not be overlooked, and should be done as frequently as possible. However, dismantling automated systems can be resource and time consuming, and therefore methods for *in-situ* calibration, characterization or validation should be explored.

## 5. Conclusions/Outlook

We have characterized the responses of a Unispec DC spectroradiometer integrated in an automated system (AMSPEC-MED) under a wide range of environmental conditions. Results show the impact of temperature, irradiance levels and illumination angle, and also the instrument settings on the spectral data acquired. Some of the effects partially cancelled out when raw spectra of each channel were divided to calculate HCRF, especially in the visible bands. For this reason and because some of the artifacts operate more strongly in the NIR, corrections had larger effects in this region than in the Visible. Though the instrumental dependencies can be also characterized as done in this work, some of these can be controlled during the design of the automated systems. Results suggest that temperature stabilization would be highly recommendable. Moreover, the estimation of spectral *DGr* is not usual in this type of systems, and further research should be done since this information is needed for the CR correction and could be applied in the use of radiative transfer models. Additional efforts should be done to correct instrumental dependencies of sensors installed in outdoors automated systems, in order to ensure quality and comparability of data, and to assure the update of the sensor models.

## Figures and Tables

**Table 1.** Summary of the calibration experiments carried out with the Unispec DC spectroradiometer. In the third column: Wp = Warm-up model. Cd = Cool-down model. St = Stable temperature.

**Experiment**	***t*_int_ (ms)**	**T (°C)**	**Recorded Spectra**
Dark current	4, 10, 15, 20, 25, 30, 35, 40, 50, 75, 100, 250, 500, 1000	Wp: [9.5, 45.4] Cd: [45.4, 24.2]	3840
Non-linearity	4, 6, 9, 11, 13, 15, 17, 18, 20, 22, 24, 25, 27, 29, 31, 33, 34, 36, 38, 71, 105, 139, 172, 206, 240, 273, 307, 341, 375, 408, 442, 454, 476, 509, 543, 577, 610, 644, 676, 741	St: [22.7, 23.9]	419
Temperature dependence	190, 283, 376, 469	Wp: [13.9, 46.1] Cd: [46.1, 25.6]	1102
Spectral calibration	7	Wp: [15.6,48.3] Cd: [48.3,18.3]	430
Cosine directional response	400	St: [26.4,29.3]	200
